# Policy vs. practice in sport and climate change: the perspectives of key actors in global sport and international development

**DOI:** 10.3389/fspor.2024.1297739

**Published:** 2024-02-29

**Authors:** Adam Ehsan Ali, Rob Millington, Simon Darnell, Tavis Smith

**Affiliations:** ^1^ School of Kinesiology, Western University, London, ON, Canada; ^2^ Department of Kinesiology, Brock University, St. Catharines, ON, Canada; ^3^ Faculty of Kinesiology and Physical Education, University of Toronto, Toronto, ON, Canada; ^4^ Department of Sports Studies, Bishop’s University, Sherbrooke, QC, Canada

**Keywords:** Sport for Development (S4D), Sustainable Development Goals—SDGs, sport policy, sport and sustainability, sport and climate, Sport for Development and Peace (SDP)

## Abstract

Despite widespread, scientifically supported recognition of the scope of the climate crisis, and policies in place connecting sport to sustainable development, there remain concerns that the environment and climate change are rarely acknowledged within SDP activity and that even when they are, it is unclear how such policies are implemented, and to what effect. This raises the question of how and why the climate crisis and the attendant relationships between sport and sustainable development are understood and operationalized (or not) by stakeholders within the SDP sector. In this paper, therefore, we explore various perspectives and tensions around the environment and climate crisis within the SDP sector. To do so, we draw on interviews with SDP policy-makers (primarily from the United Nations and the International Olympic Committee) and SDP practitioners living and working in the global South in order to gauge the place of the environment and climate change in their everyday SDP policy-making, programming and practices. Overall, the data shows that while SDP stakeholders recognize the urgency of the climate crisis, the need for action, and the policy agenda linking sport to sustainable development, significant barriers, tensions and politics are still in place that prevent consistent climate action within SDP. Policy commitments and coherence are therefore needed in order to make climate action a core feature of SDP activity and practice.

## 1 Introduction

Society has been brought to “the brink of irrevocable damage that only swift and drastic action can avert” (1). Such was the warning offered within the sixth and final assessment report of the United Nations (UN) Intergovernmental Panel on Climate Change (IPCC). The report cautioned that there is now little chance of keeping global temperatures below 1.5 C of warming above pre-industrial levels by 2030, as agreed to in the 2015 Paris Climate Accord (2), with numerous locations around the world having already warmed 2 C over the last century (3). Such warming temperatures increase the likelihood of more frequent and pronounced heatwaves, droughts, floods, ecosystem destruction, food insecurity and internally displaced peoples. Further, the effects of this changing climate will continue to impact marginalized peoples, developing communities, and island nations first and more profoundly than others, making climate change a clear matter of international relations and development (4).

Despite these warnings, the report also offered a note of hopefulness. Hoesung Lee, the chair of the IPCC, noted “This synthesis report underscores the urgency of taking more ambitious action and shows that, if we act now, we can still secure a livable sustainable future for all.” (1). Such a future is possible through re-imagining and re-structuring various sectors of society and doing so in ways that reconsider our relationship to the natural environment. In his assessment of the report, UN secretary General António Guterres’ made clear the need for this kind of expedited and comprehensive action, stating that “this report is a clarion call to massively fast-track climate efforts by every country and every sector and on every timeframe. Our world needs climate action on all fronts: everything, everywhere, all at once” (1).

These calls for change have implications for every aspect of human activity, including efforts within the global Sport for Development and Peace (SDP) sector. In recent years, SDP has grown in both size and scale, now comprised of a diverse body of organizations, including private, public and non-governmental, that all leverage the “power of sport” in various ways to respond to a range of development challenges including (but not limited to) HIV/AIDS education, gender equity, and economic development [see (5)]. Most recently, SDP has been directly connected to *sustainable* development, a concept which, among other issues, prioritizes environmental awareness, protection and remediation strategies that can help stem the tide of climate change. A recent policy brief from the UN Department of Economic and Social Affairs (UNDESA), for example, made clear these connections, noting that sport can play a key role in raising awareness of, and influencing behaviors towards climate action, making it a “low-cost” and “high-impact solution” to the climate crisis (6). The report outlined four ways in which sport can be mobilized in the fight against climate change: as a “broad social platform” for influencing attitudes about climate change, with a range that “extends to almost all geographic areas and social backgrounds”; by playing “an important role in educating and raising awareness towards global warming and…environmental issues, including promoting a healthy, sustainable lifestyle”; as a tool “to reach sustainable development, including addressing global warming”; and through “the incorporation of sustainability standards in the sport industry (that) can have a ripple effect, contributing to sustain­able production and consumption standards in other industries” (6).

Overall, however, despite recognition of the scope of the climate crisis, and policies in place connecting sport to sustainable development, there remain concerns that the environment and climate change are rarely acknowledged within SDP activity and that even when they are, it is unclear how such policies are implemented, and to what effect (7–9). Indeed, the UNDESA policy brief made note of these very concerns, remarking that even “when commitments are in place, policies and initiatives sometimes lack hard targets, mechanisms for control, a sense of urgency and/or a coherent and comprehensive strategy” (6). This raises the following question: how are the climate crisis and its attendant relationships between sport and sustainable development understood and operationalized (or not) by key SDP stakeholders?

We address this question by analyzing various perspectives and tensions around, and between, the environment and climate crisis within the SDP sector. To do so, we draw on interviews with SDP policy-makers (primarily from the United Nations and the International Olympic Committee) and SDP practitioners living and working in the global South in order to gauge the place of the environment and climate change in their everyday SDP policy-making, programming and practices. Overall, the data demonstrates that while SDP stakeholders recognize the urgency of the climate crisis, the need for action, and the policy agenda linking sport to sustainable development, significant barriers, tensions and politics are still in place that prevent consistent climate action within SDP. Policy commitments and coherence are therefore needed to make climate action a core feature of SDP activity and practice.

The paper proceeds in five subsequent parts. In the next section, we summarize some key previous studies that contextualize the complexity and challenge of prioritizing the environment and climate crisis within SDP. This is followed by a description of the theoretical framework and methodology, respectively. The results are then provided, broken down into three categories, before the discussion/conclusion is offered.

## 2 Policy (in)coherence in sport and sustainable development

The United Nations’ 2030 Agenda was released in 2015 and built around the 17 Sustainable Development Goals (SDGs). The SDGs purported to highlight the importance of the environment and the climate crisis in the struggle for global development in the 21st century. Notably for global sport and the SDP sector, Article 37 of the SDG's preamble recognized and defined a role for sport directly, calling sport an “important enabler of sustainable development,” a statement that has afforded organizations and stakeholders across the SDP sector an opportunity to conceptualize, frame and structure their work in the service of sustainability (10). The UN also detailed sport's contribution to each of the 17 Goals, including: “ensuring availability and sustainable management of water and sanitation for all” (Goal 6); “ensuring access to affordable, reliable, sustainable and modern energy for all” (Goal 7); “ensuring sustainable consumption and production patterns” (Goal 12); and “combatting climate change and its impacts” (Goal 13) (11).

From the outset, the SDGs were lauded by some for prioritizing the environment, but also criticized for their lack of focus, precision and coherence. For instance, in an article entitled “The SDGS should stand for senseless, dreamy, garbled,” economist William Easterly [(12), np] encapsulated such a critical view:

Unlike the MDGs, the SDGs are so encyclopedic that everything is top priority, which means nothing is a priority: “Sport is also an important enabler of sustainable development.” “Recognize and value … domestic work … and the promotion of shared responsibility within the household.” It’s unclear how the U.N. is going to get more women to play soccer and more men to do the dishes.

Easterly's critique is instructive for this paper as it shows that the act of simply recognizing the environment and climate change in the SDGs can be insufficient in actually making the environment and climate change a priority for policy makers and programmers because there are a host of factors that complicate the successful pursuit of any of the SDGs. Previous research shows this to be the case in sport. Lindsey and Darby (13), for example, argue that positioning sport to contribute to specific SDG targets often lacks an understanding of the causal mechanisms needed to achieve outcomes in particular national and local contexts. They also suggest that the balance between individual empowerment and structural transformation is too often overlooked, and that many “upstream” factors beyond sport (such as infrastructure, poverty, urban planning, and the obsession with organized sport) can undermine or counteract sport's SDG impacts.

Given this, Lindsey and Darby (13) concluded that the expanded scope of the SDGs requires an identification of not only the synergies, but also the incoherencies between policy and implementation, and the significance of policy sectors beyond sport. Similar tensions have been documented within the analysis of SDP national-level policies (see 14–17). What all of these studies have in common is that they show how sport-focused sustainability policies, and primarily the SDGs, are beholden to local context, organizational priorities, synergies with other policy sectors, and available resources. Indeed, influential factors extend even further when analyzing the environment specifically. In analyzing SDP and the environment, the issue of scale also needs to be considered to account for the ways in which sustainable development and environmental objectives of SDP are unique across different programs, organizations, and places (8). The prioritization of economic growth over environmental issues, and the lack of connection between SDP and external public policy areas, also represent major challenges (8), as do the differential impact of climate change on women in SDP programs (18) and the influence of the extractives industry who implement SDP initiatives in Indigenous communities (19, 20).

Given the complexity of the factors noted above in shaping the climate crisis, combined with its temporal urgency, lack of central governance and oversight, incongruence with global economic and political systems, and related conflicts in short- and long-term interests and benefits amongst public and private sectors within these systems, the relationship between climate change and SDP policy and practice fits the characterization of a wicked problem (21). The challenges of understanding, adapting, and responding to climate change are often met with uncertainty and contention amongst the numerous diverse stakeholders within overlapping societal sectors and areas (22). Within such a landscape, Head (22) identifies inclusive discussions amongst stakeholders with broad areas of expertise, from the scientific to the practical, as necessary to help build planning and problem-solving capacities (p. 673). As such, one way to address the incoherencies in sport and sustainable development policy in order to build such capacities within the SDP sector’s response to the climate crisis is through identifying, and putting into conversation, the perspectives of a diverse array of experts in this area.

Given both these tensions and opportunities, our objective in this study was to make sense of the challenge of addressing and prioritizing the environment and climate crisis in and through the SDP sector, and the challenge of moving from the SDGs as a policy agenda to climate action in SDP. More specifically, we aimed to fill a gap in the above literature by investigating where the environment and climate crisis stands within the complex policy terrain of the SDGs and the SDP sector, for both policy-makers and practitioners and why these issues have not been clear priorities; what SDP policy-makers and practitioners view as barriers to mobilizing sport in pursuit of environmentalism; and what needs to be addressed in order to prioritize climate issues and achieve policy coherence between sport and the SDGs. To address these questions, we employed a theoretical framework built around insights and understandings of policy (in)coherence, particularly with regards to sustainable development.

## 3 Theoretical framework

For our purposes, we employed the notion of policy coherence, understood as a dialectic: “On the negative side, it means the absence or removal of incoherencies, i.e., of inconsistencies between and the mutual impairment of different policies. … on the positive side, it means the interaction of policies with a view to achieving overriding objectives” Ashoff, as cited in (13). David Mosse (23) argues that policy coherence can often be divided into two largely competing perspectives: an instrumental view that sees policy as rational problem solving that should directly shape how development is done, and a critical view that “sees policy as a rationalizing technical discourse concealing hidden purposes of bureaucratic power or dominance which are the true political intent of development.” While recognizing that the latter is important, for this paper we were primarily concerned with the former, that is in trying to understand how, whether, or to what extent the SDGs lead to integrated or focused views and practices around climate responses and environmentally sustainable development in SDP. In this sense, policy coherence is intimately tied to the connectedness (or lack thereof) between policy and practice. As Mosse (23) elaborates, “Despite the enormous energy devoted to generating the right policy models, strangely little attention is given to the relationship between these models and the practices and events that they are expected to generate or legitimize in particular contexts.” He suggests that the focus on the presumed unintended “gap” between policy theory and practice obscures the actual reasons and activities that make policies implementable or not (23).

Indeed, this kind of tension has been proposed as nearly endemic to policy; while policies are written in order to solve problems, the implementation of policies is regularly left to those facing the problems themselves and, therefore, those who are most likely to lack the capabilities necessary for successful implementation (24). In turn, the more of a gap there is between the aims of a policy and the capabilities needed to act on it, the less the chances of successful prioritization and effective implementation (24). While policy coherence can be improved with stronger government involvement (25), even in cases where policies are coherent at the level of objectives, the associated instruments and in particular the implementation practices, can lead to policy conflict (26). Somewhat ironically, then, when the stakes of policy are highest, such as with climate change, and the aims of policies become further reaching, the chances of effective implementation reduce even further.

Such insights into the relationship between policy, coherence and practice have led scholars like Mosse (27) to ask whether good policies are actually implementable. Mosse's ethnographic analyses have led him to conclude that practices of international development are in fact rarely driven by policy and that “good policies” more often build political support and legitimacy rather than pathways to successful implementation or prioritization on the ground. In turn, development practitioners are motivated primarily to promote a notion or appearance of policy coherence, because it is always in their interest to “maintain coherent representations of their actions as instances of authorized policy” (27). In this regard, tensions and dilemmas between policy and practice in international development, inclusive of the SDP sector, are nearly fundamental.

Overall, we employed this kind of theoretical framework in order to investigate and account for the similarities and differences between policy makers and practitioners when it comes to understanding the place of climate change within international development, global sport, and the SDP sector. It also helped to explain and account for why, given all 17 SDGs, SDP programs and practitioners still tend to focus on those SDGs that are not environmentally focused.

## 4 Methodology

The research reported here is based on semi-structured interviews with eight policy-makers and seven SDP practitioners. These interview participants were identified using purposive and snowball sampling. Following Neuman (28), purposive sampling can be utilized when information is required of specific, specialized, or particular cases. Given that we were less concerned with the generalizability of results, and more with specific information from specialized groups and individuals, purposive and subsequent snowball sampling was fitting [see (29)].

Policy-makers were identified through a literature review, and from the organizational websites of the Commonwealth Secretariat, the International Olympic Committee (IOC), United Nations (UN), the UN Development Programme (UNDP) the UN Environmental Programme (UNEP), and the UN Educational, Scientific and Cultural Organization (UNESCO). Organizations and/or individuals were contacted based on their involvement in creating and/or supporting SDP policy documents and/or programs. SDP practitioners were identified through a search of organizations from the site sportanddev.org, as well as a general internet search. Direct involvement as an SDP organization within the last five years was the primary inclusion criteria for contact. In total, 69 individuals or organizations were contacted in the policy-maker group, and 23 in the practitioner group, with fifteen participants agreeing to an interview. All the participants involved garner a level of authority to speak on the issue of SDP and climate change, and many have had a hand in writing impactful, global SDP policy. See Table 1 for an overview of the research participants.

**Table 1:** List of interview participants.

Identifier	Organization
Practitioner A	Leading Youth Sport and Development, Togo
Practitioner B	Simama Africa, Kenya
Practitioner C	Angaza Sport and Development, Kenya
Practitioner D	Amani Kibera, Kenya
Practitioner E	Sportanddev.org
Practitioner F	Inuka Direct, Kenya
Practitioner G	Kenyatta University, Kenya
Identifier	Organization
Policy Maker A	United Nations Development Program
Policy Maker B	Commonwealth Secretariat
Policy Maker C	International Olympic Committee
Policy Maker D	United Nations Environment Program
Policy Maker E	United Nations Educational, Scientific and Cultural Organization
Policy Maker F	United Nations Framework Convention on Climate Change
Policy Maker G	United Nations Environment Program
Policy Maker H	United Nations Sustainable Development Goals Fund

Multiple individuals from each organization were contacted, and through snowball sampling, we were often referred to an organizational representative in a managerial position. One policy-maker was interviewed from each of the Commonwealth Secretariat, IOC, the UNDP, UNESCO, and the UN Framework Convention on Climate Change (UNFCCC), two from the UNEP, and one participant had worked in a consultancy role with a range of UN organizations. Policy makers were from a variety of backgrounds and lived and worked in either Western Europe or the United States. Each of the practitioners worked for an SDP organization initiated and operating in sub-Saharan Africa, with one in Western Africa, five in Eastern Africa, and one in Southern Africa. Six of the organizations represented can be classified as “sport-plus” SDP initiatives [see (30)], with a focus on sport (predominantly football) as a vehicle to promote positive social and human development ambitions. The seventh organization can be classified as a “plus-sport” initiative, where the organizational focus was on humanitarian and developmental outcomes, and sport was used in a supplemental capacity.

Semi-structured interviewers allowed for directed, yet open and free-flowing conversations, and enabled the participants to speak freely about their experiences (31). Interviews lasted between 30 and 60 min and were digitally recorded. Author 1 conducted all interviews with the policy-makers, and Author 2 conducted all interviews with the practitioners. Interviews were transcribed verbatim by a third party, and participants were provided an opportunity to review the transcripts and make any amendments or redactions. Approved transcripts were analysed using the qualitative data analysis program NVIVO, to develop coding schemes, link data and concepts, and reinforce the non-linear nature of qualitative analysis (32).

The interviews were analyzed in the tradition of critical discourse analysis (CDA). In this methodology, discourses reflect and reproduce social relations and actively construct social realities (33). CDA is commonly used within the social sciences to explore how discourses are structured, reflect broader “common sense” understandings of a particular topic, and reveal underlying power dynamics (34). We followed Fairclough's (35) five steps of analysis for CDA: exploring the social problem to be studied (e.g., how do SDP policy-makers and practitioners understand the role of sport in development?); identifying the social relationships (e.g., between policy and practice); considering whether the social relation “needs” the problem (e.g., is sport a necessary tool of development?); analyzing the discourses for broader relations of power (e.g., neo-colonial power dynamics), and engaging in reflexivity to evaluate the critique offered (e.g., what assumptions are being made?). The most discursively significant three themes are discussed in the next section.

## 5 Results

Three major themes emerged from the results of our interviews with policy-makers and practitioners that help to explain the challenges of prioritizing the environment and climate change within the sport and sustainable development policy space and translating sustainability policies into climate action. The first was that despite the fact that policy-makers and SDP programmers all agreed that sustainable development is an important consideration for SDP programming, competing definitions of sustainability and the place of climate change therein, make action challenging. The second theme revolved around barriers to implementing sustainable development policies into practice, specifically whether the environment could (or should) be prioritized within SDP, and concerns that the complexity of sustainability overall tends to paralyze meaningful climate action in the SDP sector. The third theme was the notion of policy coherence, focusing on the challenges of policy making as well as the pitfalls of a top-down, global approach. We describe each of these here.

### 5.1 Defining sustainability in SDP

There was a clear agreement amongst our interviewees that the environment is of increasing importance within sport and the SDP sector, and that SDP programs can make a positive contribution towards sustainable development, including a focus on the environment and climate change. While this view was shared by both policy-makers and practitioners, it was complicated by the fact that there were differing perspectives on what sustainable development entails, and even the threat posed by climate change. On the one hand, for policy-makers we interviewed, climate change was largely viewed as an existential crisis, and of crucial importance: this group tended to position development work at a crossroads, and pointed to the historic and pressing importance of incorporating sustainability within the sector. As policy-maker A noted:

*It’s quite a historic pivotal moment the next few decades really as to whether we can make this transformation to sustainable development so it’s becoming of course an existential crisis… last year for example was irreparable emissions and trends of biodiversity loss and extinction of about one million species… and so the role of the UN to help, continue to you know analyze those global trends, set the global agenda, highlight those continuing challenges*.

For policy-makers, given the profound threat posed by climate change, the SDGs were particularly useful for broadening the notion of sustainable development to include an awareness of the environment and climate change alongside economic and social development. Such an expansive definition of sustainability was helpful and productive; policy-maker B noted the importance of an all-encompassing understanding of sustainability, defining sustainable development as:

*the interconnectivity of economic, social and environmental development and I think that is quite, absolutely and critically important because isolating one of those three areas is not going to lead to sustainability and the sort of the second component…is not sacrificing the sort of equality and the level of equality of access to peace and prosperity of future generations for the benefit of this generation exclusively…It needs to be a focus both today but not at the, at sacrificing future generations*.

This view of sustainable development as encompassing or connecting the environment with the social and the geographic was shared by some practitioners, but in notably different ways. For this group, the expansive definition of sustainability was still laudable but also introduced a level of complexity that was challenging for those working or operating at a comparatively grassroots level or actually tasked with implementing programs. Some spoke directly to the complexity of the term, and the challenge of needing to account for so many different components which had the effect of expanding the root of sustainability beyond the environment and climate change:

*In everything you are trying to do if you want to make that sustainable you need to understand the environment, you need to understand the person and you need to understand everything that’s around that person. I think it’s a little bit more complex but sustainability for me it’s that. It’s you want to create change but if you want to make that sustainable the first thing you have to do is to understand the environment and sometimes it’s a little bit complex.* (Practitioner A)

Others, including policy-maker C, viewed the ever-shifting definition of sustainability as almost problematic or at least confusing, because it created a moving, shifting target comprised of too many elements. In other words, the SDGs represented the challenge of incorporating so many categories beyond the environment. The resulting focus on ‘everything’ in the SDG framework made it difficult to focus on anything:

*Well initially as you know by now after being bombarded with information, I suppose in the beginning the whole theme was about environment. Then it mutated into environmental sustainability and then it mutated into sustainability including everything from gender equality to flora and fauna preservation*.

An implication of this was that although the environment was seen as important by SDP practitioners and climate change was understood to pose a threat, sustainable development sometimes came to be associated more with the sustainability of the SDP sector itself, and of particular SDP programs. For example, practitioner B was clear that sustainability had become about how to make sure that their program continued running, more so than striving to ensure that life on earth could be sustained into the future:

*I define sustainability as how… can we make some of the programs that we are doing to be continuous in a way that they can be adaptable to the changing environment, changing season or maybe even to the changing situations of where we are? That’s my understanding*.

A tension here then, was evident between pursuing the sustainability of life on Earth and the sustainability of development programs themselves, and this tended to be divided between policy-makers who worked to set an agenda and programmers who worked to keep projects going. It became clear to us that policy-makers tended to focus on and advocate for relatively broad sustainability priorities, albeit from the perspective of the global North. In contrast, practitioners, living the geo-political realities of scarcity and unequal access to resources viewed sustainability as the challenge of keeping their programs going:

*I think that most sport for development programs very much operate on an individual level so they don't necessarily tackle them… at worst you could say they reinforce… the systems and structures that create some of the problems in the first place. You know it could be sort of a neoliberal type model that focuses on the individual without tackling you know the systems of equality and inequality that result in that happening in the first place…I'm not saying we don't need the interventions. We need the interventions but I think we also need a bit of a social justice approach and maybe a bit of an activist approach in some ways and you know that’s difficult because that cozy relationship between funders and you know let’s say implementers gets challenged.* (Practitioner C)

In this sense, while the SDGs might—at least in the best-case scenario—serve to connect issues of inequality and unequal development to the politics of climate change and struggles for environmental justice, we found that in practice, SDP practitioners viewed the SDGs and sustainable development as overly broad and complex, and climate change and the environment as subordinate priorities, especially relative to the sustainability of their programming. In other words, environmental notions of sustainability were often understood as second-order priorities compared to more general social or economic sustainability. We explore these tensions further in the next two themes.

### 5.2 Priorities in SDP

Following the tensions regarding the definitions of sustainability, the second theme that emerged from the interviews was the set of barriers to implementing sustainable development policies into practice, specifically whether the environment could (or should) be prioritized within SDP. While there was a desire among the interviewees for SDP to contribute to environmental protection and the climate crisis and evidence that policy-makers prioritized sustainable development and climate change, there was disagreement regarding the extent to which the climate could be prioritized within SDP programming. These shifting or dynamic priorities were illustrated by some policy-makers, who made it clear that sustainable development should be *more* of a priority within the global South than in the global North, as climate change posed a more direct and immediate threat to lower and lower-middle income, island nations, and developing polities:

*… we all know that environmental sustainability is on top of the agenda only in a few countries in the north so far,…, so that is also reflected in the grassroots sphere. But… young people in Africa and all over the world agree now that climate change is the biggest issue so that has changed. It wasn't the case until two years ago.* (Policy-maker D)

This shift in priorities might suggest that climate change would be a priority at the delivery or grassroots level of SDP. In contrast, however, several of the SDP practitioners that we interviewed were quick to highlight that climate and the environment were difficult to prioritize (while still sharing a belief in the importance of sustainability), particularly in the short term, given the range of development issues to which SDP is connected, and the global breadth and scope of other sport-related issues such as racism. Overall, the perspective put forth by practitioners was that climate change and the environment needed to be integrated into the broader ethos or conduct of SDP projects rather than positioning it (the environment) as a single issue (to be prioritized or not). The following quotations are illustrative, pointing to the challenges of prioritizing the environment in SDP programs.

*It is here every day and the challenges that we face are, they are here for a long time and we need to deal with them on a consistent basis … I think sport for development organizations should employ environment for sustainability as one of the key projects that they do (but) it just should be something that is continuous and …on a short-time basis or a seasonal project.* (Practitioner D)

*In many ways I feel like sports for development is a catalyst for general social change as well as in economic empowerment; things like growing young talents and the way they inculcate, include other aspects of development; things like gender… leadership… these are good things that are currently being rolled out in this sport for development world but… there is also glaring things in sports that sports for development does not necessarily or completely cover… like environmental conservation, the conscience of conserving sustainably utilizing our environmental resources. Things like racism, this is more of a global thing that… I would want it to come out… more deliberately in future sports for development program roll-outs.* (Practitioner E)

Our interpretation of the tensions illustrated here is that practitioners are identifying the same challenges that have long been discussed in critical studies of sport–for–development: that it is difficult to reconcile the desire for short-term demonstrable outputs with the need for long-term commitments to more complex and harder-to-measure outcomes, and that balancing the need and desire to promote skills (e.g., sport-specific skills, interpersonal, leadership and life skills) with addressing systemic issues like environmental sustainability or racism leaves practitioners in difficult positions.

For some policy-makers, the lack of prioritizing of climate and the environment within the SDP sector was frustrating, as it signalled a view from SDP organizations and programs that climate was someone else's responsibility. Somewhat ironically, these policy-makers suggested that it was the lack of an interconnected or holistic approach to sustainable development that allowed such viewpoints to take hold, even though in other parts of this research, it was precisely the broad definition of sustainable development that allowed the climate and environment to be de-prioritized. Either way, the following quotation from policy-maker E encapsulated this frustration:

*So, you know people were saying oh, we have to focus on water, and we have to focus on this and that and oh, but they didn't really have this kind of holistic thing…. So it was clear that there was no systemic approach to doing things differently and there was no, you know there was no idea really to start switching. We actually heard from one sports organization saying ‘but climate is not our duty’ you know like ‘we're not here to solve climate, we're here to stage really interesting and exciting events so that people come, have fun and then they go.’*

Similarly, policy-maker F argued that the ability to choose to ignore the climate crisis in sport and SDP, or at the very least to determine its priority status on a selective basis, has come to an end. There was a recurring sense that sport and the SDP sector need to acknowledge their position in, and the implications of, the climate crisis and that though this shift is happening, the urgency of the climate crisis means such a shift needs to happen more quickly than it currently is.

*I think in the hierarchy of needs it’s often kind of nice to have rather than must have but I think that’s changed and I think it’s changing quickly because the impact of the environment on cricket pitches, golf courses, you name it, it’s going to be significant and so I think in the transition that we're starting to see it’s going to lead to a greener and cleaner economy. Sport just needs to get its act together and move faster and not pretend it’s someone else’s problem*.

At the same time, both policy-makers and practitioners suggested that the challenge of prioritizing the environment and climate change within SDP policies and practices resulted from a viewpoint that the connections between sport, the environment and climate were relatively recent and, therefore, conceptually and practically under-developed. Both groups felt some degree of trepidation in grappling with how to approach environmental sustainability goals, given the scale, scope, and complexity of the climate crisis. For policy-makers, this created problems in designing SDP-related policies that would be intelligible, implementable and effective. For example, one policy-maker with direct experience in designing and disseminating sustainability policies for sport and SDP organizations suggested that the effective response to the crisis through policy could overwhelm practitioners without the technical knowledge required to address sustainability issues adequately. They stated,

*… in terms of climate they weren't really knowledgeable or ready to take on anything. We did see that appetite they were like we would like to do something but we don't know how and so we convened about 30 to 40 different organizations*

*…people were saying we can't just have a piece of paper cause it says nothing, we don't know how to do it. I think in my first version of the policy initiative I went through the scopes and things and categories and I think when they saw it, they were completely intimidated so I shared it with maybe 60 different sports organizations and nobody responded. Then I asked for feedback and that was interesting because people then responded yeah, but this is too complicated.* (Policy-maker E)

Thus, for policy-makers, the complexity of climate change and sustainable development was a central challenge in designing effective policies and in soliciting ‘buy-in’ from sport and SDP stakeholders. For practitioners, while lack of expertise in sustainability or climate change was a concern, the more pressing issue was a need to be sure that policy-making was grounded in evidence before they could take on such policies and adopt them into their programs on the ground. Such concerns again spoke to the relatively recent acknowledgement of sustainable development within SDP:

*Most countries accept that sport and physical activity contribute to health and that’s reflected in their policies. Whether it’s implemented, whether it’s measured well that’s a different story. But you know, there was bound to be a dearth, a lack of policies relating to sport that emphasized the environment you know or, and a lack of evidence related to sports you know impact on the environment … we don't really have that much evidence… around the ways sport can get around the frontline of crisis and improvement here and or matter of fact effecting policies* (Practitioner C)

Here, the sense was that the environment and climate crisis has not been a priority in the SDP sector because of the lack of evidence that sport and SDP can adequately respond to the crisis. The implication in this quotation is that if SDP practitioners could be sure that they knew how to contribute to the climate crisis, they would do so. However, until such time, they have to focus elsewhere. This perspective speaks to the challenges of policy (in)coherence, explored further in the next and final theme.

### 5.3 Policy coherence and the SDGs

The third theme builds on the previous two and focuses on policy coherence. In this sense, as we set out to understand and account for why the environment and climate crisis were not being prioritized in the SDP sector, we came back, time and again, to the challenges of coherence posed by the SDGs themselves. While the shift from a relatively narrow set of development issues to a more expansive view of development focused on sustainability ostensibly opened up the *possibility* of climate change and the environment becoming a priority in SDP, it also served, in some cases, to increase the difficulty of prioritization given all of the development goals posed by the SDGs. Overall, while most noted the prioritization of the environment and climate change in and through the SDGs and lauded them as a roadmap, the SDGs were also, in some cases, precisely part of the problem of environmental and climate prioritization within SDP. For example, on the one hand, some interviewees viewed the expansiveness of the SDGs as a point of direction for policy-making that was serving to prioritize the environment in SDP. As one policy-maker noted,

*I think they're hugely helpful … I think without them the world would be lost -so they lay out a map. They're our GPS for where we need to get to. There’s an interrelationship between them.So, if you look at the environmental and the ecosystems one, then the social and the economic ones: unless you have a viable planet then all the social and economic count that we've striving for if they're not underpinned by a healthy ecosystem then all of this would just topple … So, there’s such an interplay between the SDGs. So, for me I find it really helpful and I work for the UN so I probably shouldn't say this but my feeling is that it doesn't need to be religion. It just needs to be the North Star or a southern cross. It just gives people a perspective of where we're trying to get to.* (Policy-maker F)

The expansiveness of the SDGs was also viewed as helpful by some SDP practitioners because in instances where knowledge of matters of sustainability and climate change was lacking, the goals provided legitimization and guidance as what should be prioritized and the importance of sustainability. As Practitioner B noted:

*For me the SDG has been helpful because … for my organization it has helped in a way that I prioritize issues on a more, on a more sustainable way? How are the goals working? It has also helped me to narrow in some of the key important goals that I can… use in my organization. And what I have done is that first of all we've gone to the local groups in my community… to sensitize people on sustainable development*.

In this sense, and particularly for some policy-makers, while policy coherence was a challenge, it was a productive one. In other words, the SDGs offered an opportunity to promote coherence and cohesiveness because of the imperfect nature of policy-making and the potential problems posed by an ever-growing SDP apparatus. The following from policy-maker B points to such a view:

*My favourite SDG (laughs) is probably 17 … which is around policy coherence for sustainable development and indeed if you sort of look at policy coherence it's essentially around … trying to enhance synergies between different policies. But then understand, manage and negotiate trade-offs and for me- this is at the core of the SDGs and more so at the core of the interconnectivity of the social, environmental and economic … So, really for me it’s accepting that there are always trade-offs for different policy … that need to be understood and critically in this context or rather discussion the trade-offs in particular economic policies will have on environmental issues and vice versa*.

Similarly, policy-maker D noted that the SDGs offered an opportunity to promote a common approach to sustainable development through sport and the SDP sector on a united front, so as to ensure common understandings amongst policy-makers, officials, and practitioners.

*Policy coherence meaning if we want sport to be more than a side agenda, a marginal portfolio in governments misunderstood as an opportunity to refine with sports stars and to get medals then we need a critical mass at all levels. A critical mass meaning common methodologies, aggregating data, pooling resources, combining tools at all levels as I say from grassroots up to policy. Otherwise we will continue working in our respective silos and we just continue to be marginal in the policy space and we did this one by this mighty stakeholder approach so it continues involvement of all sorts of people in constructing the agendas … i.e., we have now an action plan, agreed upon consensually with the IOC, with FIFA, with all the UN organizations onboard*.

For their part, some practitioners also viewed the SDGs as a potentially useful tool in providing some degree of policy coherence. In speaking to the utility of the SDGs, practitioner C suggested that the SDGs could be the backbone of a coordinated approach to sustainable development in and through SDP:

*Well in a way it’s been useful I think to have a common framework that you know sport for development actors … would contribute to some sort of coordinated action and frameworks like measurement frameworks but also implementation frameworks. So I think that’s positive to sort of ensure some level of coherence. And although that’s quite difficult to do in any sector, in the sport for development and peace sector that’s you know often quite civil society driven … with a massive diversity of approaches—it could be sport and environment, it could be sport and health, it could be sport and disability—it makes it quite difficult to standardize anything but I think they are useful*.

That said, despite the policy benefits ascribed to the SDGs, there remained a sense among some of our interviewees that climate and environmental issues were beyond the purview of the sport and SDP sectors and that many in the field tended to think of these issues as ones to be taken up by environmental experts, rather than integrated into a coherent policy framework of sustainable development. In particular, the challenges posed by such a shift and from 8 to 17 goals, were noted by policy-makers we interviewed, such as in the following quotation:

*…environmental issues, notably climate change, are not usually specifically prioritized in policies and plans concerning sustainable development. This is [regrettable] especially so in most developing countries where environmental challenges in general and climate change in particular are not integrated fully in the development policy context. In many cases governments in developing countries believe that their Ministries of Environment or Climate Change are capable of tackling all aspects of the environmental scenarios and the impacts of climate change. Accordingly, think tanks and civil society organizations find themselves having to devote a large part of their efforts and resources on highlighting the imperative of integrating climate change and environmental issues into the fabric of the national development policy formulation and implementation processes.* (Policy-maker G)

The range of different actors brought into the field, at times with competing perspectives and objectives was a challenge posed by the broad sustainable development agenda. Similarly, others viewed the depth and breadth of policies related to sport and sustainable development as a problem in meeting the specific and contextual needs for development programs. As policy-maker F expressed, the scope of the SDGs is hard to embrace or digest:

*The SDG I think is a huge pill to swallow. It’s like you're giving people like an eight-course dinner to try and it’s overwhelming. So, for me I've always ever seen it as a buffet that you can choose from depending on what you're interested in and I think that’s a better frame cause if you're trying to make the SDGs famous then I don't think that’s the right approach. I think there are many tools of leaders that you can pull to really holistically make a difference in the world and if you're a systems thinker then I think that’s fine- but I don't think people think about causes in a systems framework and nor should we expect them to*.

Other policy-makers also offered critiques of the SDGs and attempts to streamline and standardized development objectives. For some in this camp, the SDGs were helpful, but the point about the expansiveness of the goals as noted above, compromised coherence. Policy-maker F, for example, viewed the number of SDGs are contributing more to confusion than coherence.

*I think that’s helpful. I mean I think what wasn't helpful was having 17. It’s more like they should have had five or ten max but- the member states couldn't agree so they just dumped a whole lot of things in there that I just think just make it less easy for people to navigate so-people can barely remember three things; five is a struggle, ten is impossible. 17 you're not making it easy for folks but if you're going to have to do 17—so for me it’s more the clarity of the intention- rather than the ability of the content but I think they're a big improvement on the MDGs. … I think they've been really useful.*.

Overall, the interview results indicate that the SDGs, when viewed positively, were seen as a way to promote a holistic view of sustainable development within the SDP sector. At the same time, for many stakeholders, the goal and task of prioritizing the environment and climate change was actually made more challenging because of the expansiveness of the SDGs and the difficulty of integrating them into a focused, coherent vision of sustainable development for sport and the SDP sector. In the penultimate section of this paper, we discuss the implications of these results.

## 6 Discussion

In their work on policy coherence, Lindsey and Darby (13) concluded that the expanded scope of the SDGs requires an identification of not only synergies, but also the incoherencies between policy and implementation, which account for the impact of policy sectors outside of the sporting landscape. Our results illuminate not only the incoherencies themselves but also the factors that make it challenging to move from sustainability policy to climate action in SDP. These include the varied and competing definitions of sustainable development/sustainability, the relative nascency of environmental initiatives in sport and SDP, the top-down direction of SDG policy/implementation in SDP, and the interrelated focus on environmental awareness-raising. These factors shape not only how environmental policy in SDP is created, and by whom, but also reproduce the circumstances under which such incoherencies remain pervasive in sustainable development policymaking in SDP. Given these factors, we argue that while the SDGs are a useful guide for policy-makers and practitioners, they likely cannot be solely relied upon to prioritize climate action, responsible interactions with and protection of the natural environment, and sustainable development objectives within the SDP sector.

Several implications follow from this; first, the role of sustainability itself. The results demonstrated differences between policy makers and practitioners when defining and operationalizing sustainability in an SDP context, even though both groups agreed that SDP could positively contribute to sustainable development. These tensions align with Mol's (36) assertion that sustainability is primarily a global attractor that becomes a “point of orientation… for local and global flows and networks” (p. 525), even while its definition and interpretation remain nebulous and contested. While Mol derived this point through his analysis of the 2008 Beijing Olympics, here we suggest that the SDP sector may also be considered a point of convergence “for a cluster of major developments at and between different levels of social life” (36). This convergence is evident to us in that sustainability discourses produced both agreement and dissonance between practitioners and policy-makers regarding the significance, definition, and uptake of the environment and climate crisis, all of which were highly dependent on local context.

Indeed, the inclusion of economic and social forms of sustainability within the SDGs, the discursive strategies different stakeholders use to promote the idea of sustainability within SDP, and the ways in which SDP practitioners discussed strategies to maintain the sustainability of their programs, rather than the environment itself, demonstrate how different actors, institutions, and networks “articulate and include sustainability considerations and interests” (36). Considering sustainability as a point of convergence for policy-makers and practitioners also illuminates the different conditions and challenges these networks of individuals face in SDP. Policy-makers, who tend to work within relatively stable intergovernmental organizations, were privileged with the time and space to reflect on problematizing the role of sport in sustainable development. Conversely, practitioners were more concerned with ensuring that their programs, communities (and, perhaps, livelihoods), which are increasingly affected by the climate crisis, remained sustainable. This challenge makes it worth considering whether and to what degree the concept of sustainability remains important for forwarding climate advocacy and objectives in SDP and elsewhere.

A second implication to emerge from the data was that of policy coherence, particularly regarding top-down development. Lindsey and Darby's (13) reminder to pay attention to the specific mechanisms of sport for contributing to the SDGs could be seen in the hesitation both policy-makers and practitioners expressed in approaching sustainability goals through SDP work, given the complexity of climate crisis, the issue of scale in policymaking, and the relative lack of evidence-based guidance. While for some participants, the expansiveness of the SDGs was a helpful policy coherence mechanism as they acted as a common framework for practitioners and policy-makers to aspire towards, for others, the SDGs led to policy confusion by having to navigate the sheer breadth of the agenda. It was also clear that a desire to respond to the climate crisis in and through SDP was impeded by specific barriers often not recognized in policy: limited resources and support, increasing competition for limited funding opportunities between SDP organizations, and a lack of clarity surrounding measurement, monitoring, and evaluation all acted as significant barriers to implementation. It also needs to be noted that both groups interviewed in this study agreed that top-down policies, even if undergirded by national and regional politics, could still create challenges for practitioners trying to implement them within specific, localized contexts.

The resulting implementation gap evidenced in this study aligns with and largely confirms previous research on institutionalizing the SDGs through sport. For example, Charway et al's work in Ghana found that while the Ghanian government signalled its commitment to sport for sustainable development in its policy agendas, it failed to implement and measure SDG-related targets in its communities. Regional-level commitment to policy was thus a form of “symbolic implementation” characterized by a lack of direction, resources, and the prioritization of national sports teams over SDG targets, resulting in local-level challenges that included a lack of funding, a disproportionate dependence on football activities, and the neglect of frontline practitioners due to political (dis)favour and clientele-based relationships. Our findings suggest that such challenges are also present on a more macro level or international scale, in which international agenda-setting around climate issues in SDP tends to fall short of supporting climate action. Thus, there remains an “implementation deficit” in which local practitioners are left to pursue goals that make the best sense for their communities but diverge from international, national and/or regional objectives (14). In such cases, climate change continues to be relegated to a subordinate position. Put differently, while horizontal coherence of policies across different sectors is important, our results demonstrate that the complex challenges facing policy-makers and practitioners, from a lack of resources and support for evidence-based approaches to unequal relationships of power and knowledge production between national and international SDP stakeholders, make such coherence improbable under the current circumstances.

Finally, it is worth reflecting on the commonly held idea that sport's contribution to sustainable development lies primarily in raising awareness about environmental issues among consumers or individuals more broadly. While a range of studies has revealed important limitations of technocratic, individual, and/or behaviour-change approaches to environmentalism and the climate crisis (e.g., 37, 38) we recognize that such approaches may be the only way some organizations, particularly those with limited resources, are able to respond to climate issues. However, we should also question what awareness is being raised and towards what ends. In other words, if the focus of sport and the SDP sectors remains on “raising awareness” of environmental issues, we are concerned that this may de-emphasize the need to make fundamental and systemic changes to how sport and SDP are practiced towards achieving more substantive climate action objectives.

Researchers have made similar critiques in previous studies of SDP and of sports mega-events regarding the tendency to rely uncritically on neoliberal and ecological modernist logics by placing faith in the free market and emerging technological solutions to address environmental challenges in sport (39, 40). This is to say that, as Lindsey and Darby (13) note, individual empowerment (and awareness) should be pursued alongside approaches that engender social change. In other words, while the focus in this paper is on climate issues specifically, the tensions here reflect previous critiques of SDP; namely, emphasis on awareness raising and behaviour change in ways that may fail to address the structural conditions that increasingly magnify the scope of the crisis (see 41, 42).

## 7 Conclusion: pushing sustainable development policy in SDP forward

In this paper, we have explored the often-limited place and role of the environment and climate action within SDP policy and programming by drawing on interviews with policy-makers and practitioners in the sector. The interviews revealed a collective understanding of the urgency of the climate crisis amongst both groups of SDP actors and the importance of meaningfully connecting sport to sustainable development policy. At the same time, the interviews also demonstrated significant challenges in translating these intentions to action, including contested definitions of sustainability amongst various SDP actors, competing priorities within the SDP sector, and a lack of policy coherence buttressed by the breadth of the SDGs. These challenges suggest that while SDP stakeholders collectively recognize the importance of signaling sustainability as a priority within the sector, tensions remain regarding how to best implement environmental initiatives, peripherally or centrally, within SDP policy and programming. Future work in this area should look to expand the breadth of research participants beyond western intergovernmental organizations and African-based SDP NGOs. We suggest this because of the regional specificities of both the SDP sector and the particular challenges posed by climate change. Asking similar questions to SDP practitioners working in the Middle East, South Asia, and North and South America would be a logical next step to interrogate further the place and roles of sustainability across geopolitical divides. Additionally, it would help to better understand the implications of regional and international politics on the environment and climate action within the global SDP landscape. Asking similar questions of SDP practitioners and, indeed, of policy-makers from different governance and international bodies would expand our understanding of a global problem that cannot be universalized, especially given the demonstrated need to develop locally-driven programming in both realms of sport and sustainability.

## Data Availability

The raw data supporting the conclusions of this article will be made available by the authors, without undue reservation.
